# Regional myocardial edema detected by T2 mapping is a feature of cardiotoxicity in breast cancer patients receiving sequential therapy with anthracyclines and trastuzumab

**DOI:** 10.1186/1532-429X-16-S1-P273

**Published:** 2014-01-16

**Authors:** Paaladinesh Thavendiranathan, Eitan Amir, Philippe Bedard, Andrew Crean, Narinder Paul, Elsie T Nguyen, Bernd J Wintersperger

**Affiliations:** 1 Cardiology, University of Toronto, Toronto, Ontario, Canada; 2 Radiology, University of Toronto, Toronto, Ontario, Canada; 3 Medical Oncology, University of Toronto, Toronto, Ontario, Canada

## Background

Cardiotoxicity from cancer chemotherapy is an important cause of morbidity and mortality in breast cancer survivors. Left ventricular (LV) systolic dysfunction due to sequential use of anthracyclines and trastuzumab has been reported in up to 20-25% of patients. The pathophysiological changes associated with LV dysfunction have not been described non-invasively. We performed cardiac MRI (cardiac MR) with T2 mapping to assess whether myocardial edema is a feature of acute cardiotoxicity in women with breast cancer receiving sequential anthracyclines and trastuzumab.

## Methods

We prospectively recruited women with HER2+ breast cancer scheduled to receive anthracyclines followed by trastuzumab (Grp A) and patients during therapy with anthracyclines and trastuzumab who were diagnosed with subclinical cardiac toxicity (Grp B) using multi-gated acquisition scans (MUGA) based on Cardiac Review and Evaluation Committee's criteria. All patients (July to Aug 2013) underwent cardiac MR imaging at 1.5 Tesla with conventional steady state free precession cine and late gadolinium enhancement (LGE) imaging with addition of quantitative T2 mapping using a T2-prepared single-shot SSFP technique. LV volumes and systolic function were quantified and segmental T2 values for all 16 myocardial segments were obtained from basal, mid, and apical short axis views. T2 values > 59 ms were considered abnormal based on previously published data. Mann-Whitney rank sum test was used for comparisons.

## Results

**Figure 1:**
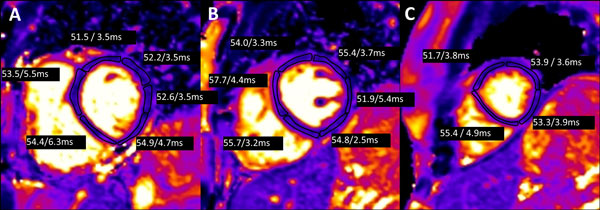
**T2 maps in for a patient pre-initiation of therapy**. The mean T2 values for each segment and the standard deviation (mean/standard deviation ms) are shown and were all within normal values. The images represent (A) basal, (B) mid, and (C) apical short axis T2 maps. LVEF in this patient was 57%.

**Figure 2:**
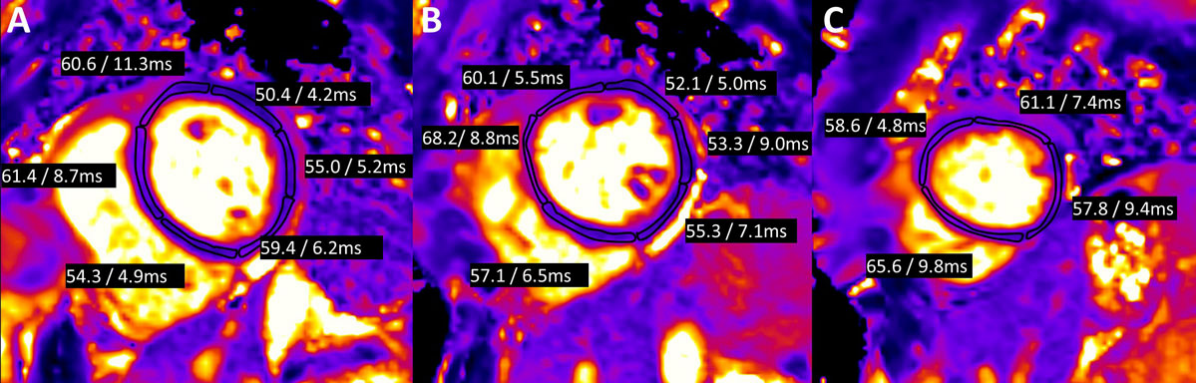
**(A-C) Segmental T2 values (mean/standard deviation) for the (A) basal, (B) mid, and (C) apical myocardial segments in a patient with cardiotoxicity**. There were 7 segments with abnormal T2 values (defined as T2 values > 59 ms). Mean T2 values of the abnormal segments was 62.3 ms. LVEF in this patient was 46% (from 63% pre therapy).

Preliminary results: Patients in Grp A (n = 6, age 44 ± 8 years) had higher LVEF than patients in Grp B (n = 3, age 50 ± 9 years) - 59 ± 7% vs. 49 ± 5%; p = 0.02. While all segments (n = 96; 100%) in patients in Grp A showed T2 values < 59 ms with a mean of 53.6 ± 2.9 ms (47.5-58.8 ms), in patients in Grp B 14/48 segments (29%) showed abnormal T2 values > 59 ms with a mean of 63.5 ± 4.2 ms (59.4-70.9 ms); p < 0.001. The remaining 34 segments in patients in Grp B demonstrated T2 values < 59% with a mean of 55.2 ± 2.3 ms. Two out of the 3 patients in Grp B had minimal mid-myocardial scar on LGE imaging. Figures 1 and 2 illustrate T2 values in a patient in Grp A and Grp B respectively.

## Conclusions

This preliminary analysis of a larger ongoing study demonstrates significant increase in segmental T2 values in breast cancer patients with cardiotoxicity compared to those pre-initiation of chemotherapy. This suggests that myocardial edema may be a feature of cardiotoxicity that results from treatment with sequential anthracyclines and trastuzumab. Whether quantitative assessment of myocardial edema early during therapy can predict subsequent development of cardiotoxicity remains to be determined.

## Funding

Grant Miller Cancer Research Fund.

